# Recent regulatory developments in EU Medical Device Regulation and their impact on biomaterials translation

**DOI:** 10.1002/btm2.10721

**Published:** 2024-10-16

**Authors:** Klaudia M. Jurczak, Torben A. B. van der Boon, Raul Devia‐Rodriguez, Richte C. L. Schuurmann, Jelmer Sjollema, Lidia van Huizen, Jean‐Paul P. M. De Vries, Patrick van Rijn

**Affiliations:** ^1^ Biomaterials and Biomedical Technology Department‐FB40 University of Groningen, University Medical Center Groningen Groningen The Netherlands; ^2^ Department of Surgery, Division of Vascular Surgery University Medical Centre Groningen, University of Groningen Groningen The Netherlands; ^3^ Quality and Safety University Medical Center Groningen Groningen The Netherlands

**Keywords:** biomaterials, biomaterials clinical translation, In Vitro Diagnostic Regulation, Medical Device Regulation, medical devices

## Abstract

We envision this work to assist researchers and medical device developers (beside other stakeholders) to better understand biomaterial‐based medical device development and its approval process proposed by the new MDR and IVDR in the European Union, as more complex biomaterials emerge, with the MDR reflecting the progress in biomaterial discoveries. Additionally, insufficient international harmonization in regulatory laws and poor‐quality data reporting contribute to the problem. This review describes the possible reasons for a slowing biomaterials translational trend observed over the past decades, focusing on the European Market, and suggests a feasible approach for biomaterials‐based medical device translation into the clinic. Suitable solutions to upgrade biomaterial translation to the clinic have not yet been provided by the field: no additional hurdles should be imposed for researchers, clinicians, the medical device industry, and insurance companies, which all should collaborate on bringing innovative solutions to patients. The new MDR and IVDR represent a substantial advancement in ensuring patient safety and reflect a major step forward in healthcare. However, they should not constrain innovation in biomaterials‐based medical device development. Incorporating reverse engineering from patient safety and a ‘safe by design’ (SbD) strategy early into medical device development might lead to a smoother and successful approval process. A solid R&D phase, with an emphasis on device safety and performance assessment, is fundamental to ensure an effective transition into the clinic. We offer an overview of the recently implemented regulations on medical devices and in vitro diagnostics across the EU, describing a shifting paradigm in the field of biomaterials discovery. As more complex biomaterials emerge, suitable regulations will be necessary to keep bringing safe and well‐performing medical solutions to patients.

**List of abbreviations**
CIP: clinical investigation plan
EMA: European Medicines Agency
FbM: function by manufacturability
FDA: Food and Drug Administration
HTS: high throughput screening
IH: in‐house
ISO: International Organization for Standardization
IVD: in vitro diagnostic medical device
IVDD: in vitro diagnostic directive
IVDR: In Vitro Diagnostic Regulation
MD: medical device
MDD: Medical Device Directive
MDR: Medical Device Regulation
MS: member state
NBs: notified bodies
QMS: quality management system
SbD: safe by design
TTC: technological transfer center
UDI: unique device identification

**Translational Impact Statement**

This manuscript intends to aid researchers and medical device developers, alongside other stakeholders, in comprehending the development and approval process of biomaterial‐based medical devices under the Medical Device Regulation (MDR) and In Vitro Diagnostic Regulation (IVDR) in the European Union. By discussing translation challenges, legislative discrepancies, and potential strategies, we aim to smoothen the clinical translation process from bench to bedside, drawing insights from recent literature to effectively guide stakeholders through the evolving regulatory landscape.

## 1 EVOLUTION OF BIOMATERIALS TRANSLATION AND THEIR CLINICAL NEED

The World Health Organization (WHO) estimates that “by 2030, 1 in 6 people in the world will be aged 60 years or over. By 2050, the world's population of people aged 60 years and older will double (2.1 billion), while the number of persons aged 80 years or older is expected to triple between 2020 and 2050.”^1^ Effective treatment of diseases, vaccines, better food preservation, and general access to healthcare are among the reasons for this increasing trend of healthy aging. A large part of this trend is connected to innovative developments in medical (implant) technology.

Throughout history, many materials have been utilized in attempts to solve medical problems.^2^ These materials are described as “biomaterials”, and are made of a wide variety of compounds, including elastomers (silicone), hard polymers (degradable and non‐degradable, e.g., polyethylene, polypropylene, polycarbonate, polylactide, and polyglycolide), acrylates (polymethyl methacrylate), fluorinated polymers (polytetrafluoroethylene), metals (cobaltchrome, titanium, stainless steel), and inorganics (aluminiumoxide, calcium phosphates). The earliest definition of a biomaterial was outlined by orthopedic surgeon Jonathan Cohen in 1967,^3^ who stated that all materials that are used as implants, with the exception of drugs and soft biological tissues, are “biomaterials.” Currently, the field addresses a biomaterial as “a nonviable material used in a medical device, intended to interact with biological systems”^4^ “in order to maintain or improve quality of life of the individual.”^5^ In 2018, during the Consensus Conference in China, the term ‘biomaterial’ was redefined as “a material designed to take a form that can direct, through interactions with living systems, the course of any therapeutic or diagnostic procedure.”^6^

The fact that the definition of the word “biomaterial” became more and more specific over the years coincides with an observed stagnation of actual biomaterials being successfully implemented into the clinic.^7^ Ren et al. categorized several stages in this stagnation by observing the historical timeline. In the first phase (1900–1967: “Age of the Giants”), the journey from bench to bedside could be as short as 2 years, leading, for example, to the modern treatment of diabetes. One of the most influential giants in the field of biomedical engineering was the “Father of Artificial Organs”, Willem J. Kolff. Kolff successfully invented the first kidney dialysis device during WWII^8^ and, later on, contributed to the design foundation of the first artificial heart. Along with W. J. Kolff came Forrest Bird with a positive‐pressure face mask used by pilots during WWII and later advanced to medical respirators still used today.^9^ Besides leading to great biomedical discoveries, the huge experimental freedom that existed in the first phase gave rise to ethical question marks. In the Transition phase (1970–1990), following the implementation of the Helsinki Declaration (1964),^9^ principal investigators in the (bio)medical field had to justify their clinical trial methodologies (how data was collected and analyzed) and take into account their patients well‐being in association to the risks of experimental therapies. As a result of this shift towards securing safety for test subjects, the governmental oversight of new treatments and technologies increased. The end of the ‘Age of the Giants’ and the large stream of approved medical products was accompanied by the start of the new, officially recognized, multidisciplinary field of biomedical engineering (BME).^10^

This new era describes the shift of research from the macro‐level of organ systems to the micro‐level of the cellular and molecular aspects of diseases. New research fields arose, such as tissue engineering, bio‐ and nanotechnology, and artificial organs, to name a few, in which developments accelerated and new giants came into the picture, such as Robert S. Langer, with synthetic polymeric scaffolds or Anthony Atala, who is working on 3D printed organs. Contradictory, the clinical translation and actual implementation of findings in these new fields were limited.^11^ A large part of this stagnation could be attributed to even more present governmental oversight about animal studies and human clinical trials and to the costs related to these investigations. For instance, an attempt to estimate the costs from current literature related to in vivo animal studies for biosynthetic polymeric nerve scaffolds added up to 55ME, which resulted in three commercially available materials: collagen, polycaprolactone, and polyglycolic acid. Costs that are related to the entire translational process from bench to bedside, can hardly be estimated because of the diversity of medical devices.^12^ Some requirements of the latter were sometimes even reported as nearly impossible to meet.^13^ Around the same time, technology was moving faster than research standardization. The first cell line of mouse fibroblast (L929) was described by Earle in 1948, whereas the cell quality guidelines with the requirements of implementing CO_2_ incubators, laminar‐flow hoods, and essential media to the culturing practice became uniform only during the 1960s after the first human‐derived cell line (HeLa) started to be used worldwide.^14^ The word ‘biocompatibility’ started to appear in the scientific jargon, leading to the publication of ‘Standard Practice for Direct Contact Cell Culture Evaluation of Materials for Medical Devices' (F813‐83) by the American Society for Testing and Materials (ASTM) in 1983.^15^ However, it was not until 1993 before the International Organization for Standardization (ISO) guidelines were introduced to the scientific community and aimed to focus researchers and stakeholders on the safety and biocompatibility of commercialized biomaterial‐based devices. This led to in vitro testing for biomaterials becoming common practice. In more recent decades, developments in biomaterials have accelerated even more.

### 1.1 Biomaterials

Clearly, as our understanding of biomaterial–cell interactions has grown tremendously, so have the biomaterials themselves become more and more complex when it comes to their design and surface characteristics. They can be classified into subcategories throughout the historical timeline, chronologically listed in Table 1. Their further categorization as medical devices and in vitro diagnostic devices are discussed in Section 3.

**TABLE 1:** Listed generations of biomaterials across the decades based on their intended purpose and characteristics.

Type of biomaterial	Timeline	Generation	Purpose
Inert	1960–1970	First	Replace damaged tissue, and provide structural support.
Bioactive	1980–1990	Second	Increase the device's effectiveness by utilizing coatings to provoke the biological reaction between the material and the host.
Biodegradable	2000–2010	Third	Combat infections arising from toxic or immunological processes by degrading over time.
Biomimetic/smart	2010–present	Latest	Mimic the host micro‐environment and aim to promote a specific cell or immunogenic response.^16^

**FIGURE 1:**
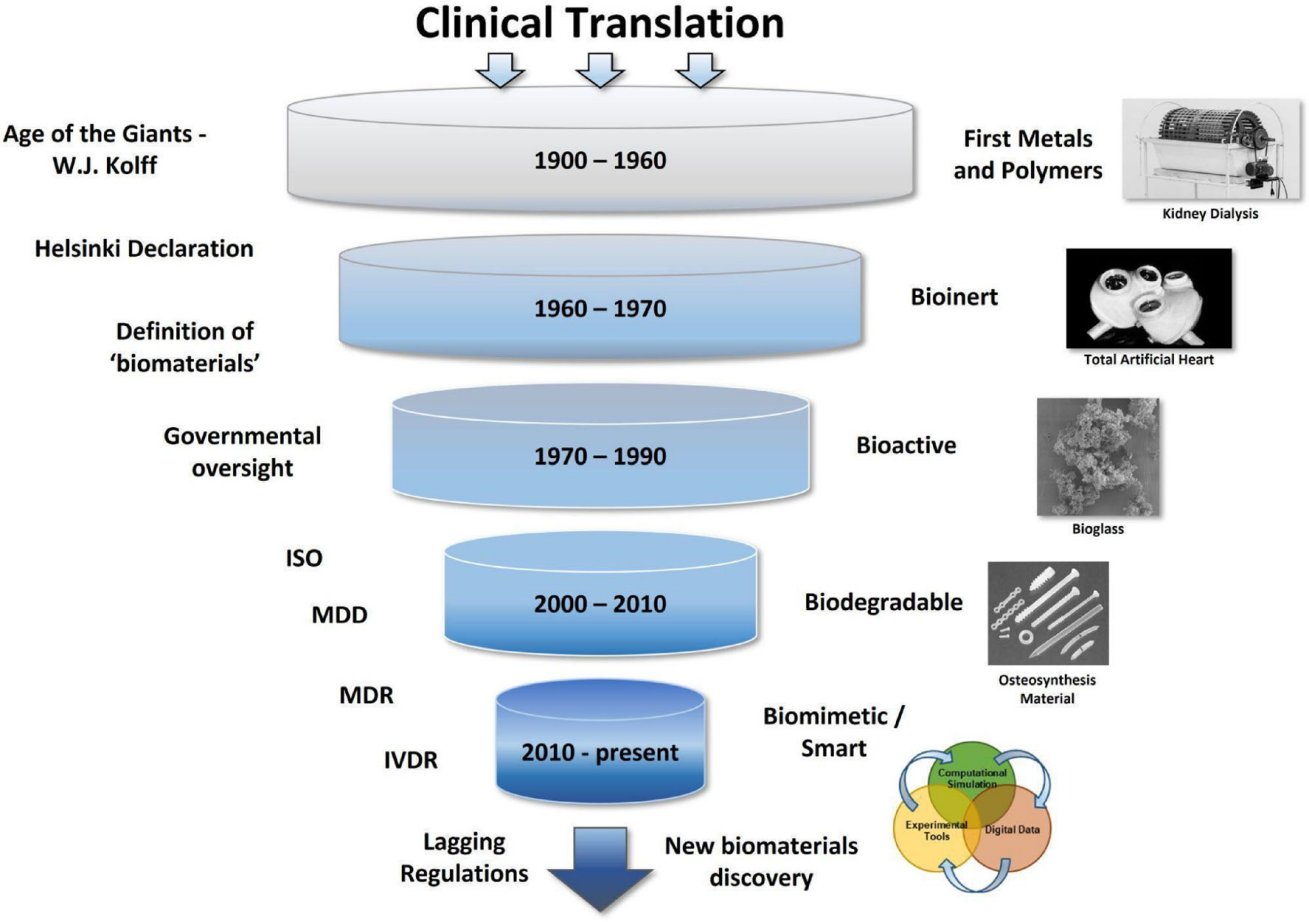
Stagnation in clinical translation of biomaterial‐based medical devices observed over time. Historical events listed in chronological order (left), accompanied by biomaterials utilized the most (right) in those eras. Increased device complexity in parallel to tightening regulations leads to a translational bottleneck.

The development of medical devices and ‘biomaterials discovery’ can be divided into two distinct phases: (1) the classical approach; making use of existing materials to try and solve a clinical problem, and (2) the new approach; designing and developing new materials to tightly match the actual needs of the clinical problem. This shift (discussed in detail in Section 2) in design thinking is starting to confront the field with standards of guidelines for good measurement and testing (ISO) that do not reflect the real situation in the human body. Moreover, designing new biomaterials means having to go through regulations from the start, a costly and slow process, resulting in a decrease in approved medical devices in the European Union. It was reported that, in recent years, large companies have been neglecting the EU when launching their devices.^17^ In parallel to the stagnating trend of translation into the clinic, there is an increasing need for (smart) biomaterials in today's healthcare. All these factors result in the increase in time‐to‐clinic, which in turn delays the clinical validation of new biomedical products and technologies.^17^ Consequently, it reduces market returns for product developers and increases the costs of new research while simultaneously decreasing the willingness of all stakeholders to invest. This downward funnel of stagnation (Figure 1) seems to become a substantial problem.

In this work, we aim to outline recent developments in biomaterials‐based medical devices, specifically focusing on their safety and manufacturability aspects (Sections 2 and 4), on the one hand, and on the other hand, we explain the important aspects of the recently implemented European Medical Device Regulation and In Vitro Diagnostic Regulation (MDR & IVDR) (Sections 3 and 4). More importantly, we identify how these two relate to one another in a clinical translational context, ultimately aiming to assist researchers and medical device developers (besides other stakeholders) in following a smoother and more successful path to clinical translation within the European market.

## 2 APPROACHES FOR BIOMATERIALS DISCOVERY

Biomedical research aims to understand human physiology and tries to provide more potent methods of diagnosis, treatment, and prevention of diseases. In compliance with ethical approvals, trials with biomaterials in human subjects can only take place at the very developed stages of testing new medical applications. In the pre‐clinical setup, testing of the new biomaterial‐based application can be performed in either in vitro models or in animal models. Once the biomaterial‐based device is tested on safety and performance requirements, it may enter the human clinical trials phase. In the field of implantable devices, biomaterials have been playing an essential role in inducing healing processes in the human body. The implantation site and medical purpose of the device should dictate the material selection and the device design to best support material‐tissue interactions and prevent implant rejection.

### 2.1 Classical approach

Retrospectively, the first types of materials used in clinical applications were inspired by nature and were based on scientific ingenuity when applied to the human body.^18^ For instance, collagen, silk, and chitosan have been widely utilized natural materials and are still being used in modern research. Collagen is the most abundant extracellular matrix (ECM) protein and is the main component of connective tissue. Its first application as a biomaterial was reported in the design of a suture called “catgut” in 1881.^19^ Silk, on the other hand, plays an important role in reconstructive and revision surgery and is employed as a surgical scaffold that resorbs over a long‐term period, supporting neovascularization and native tissue regrowth.^20^ Chitosan is known to be biodegradable, biocompatible, and non‐toxic and, over the years, has been utilized for drug delivery systems, implant coatings, wound healing applications, scaffolds, and cosmetic formulas, to list a few.^21^, ^22^, ^23^

**FIGURE 2:**
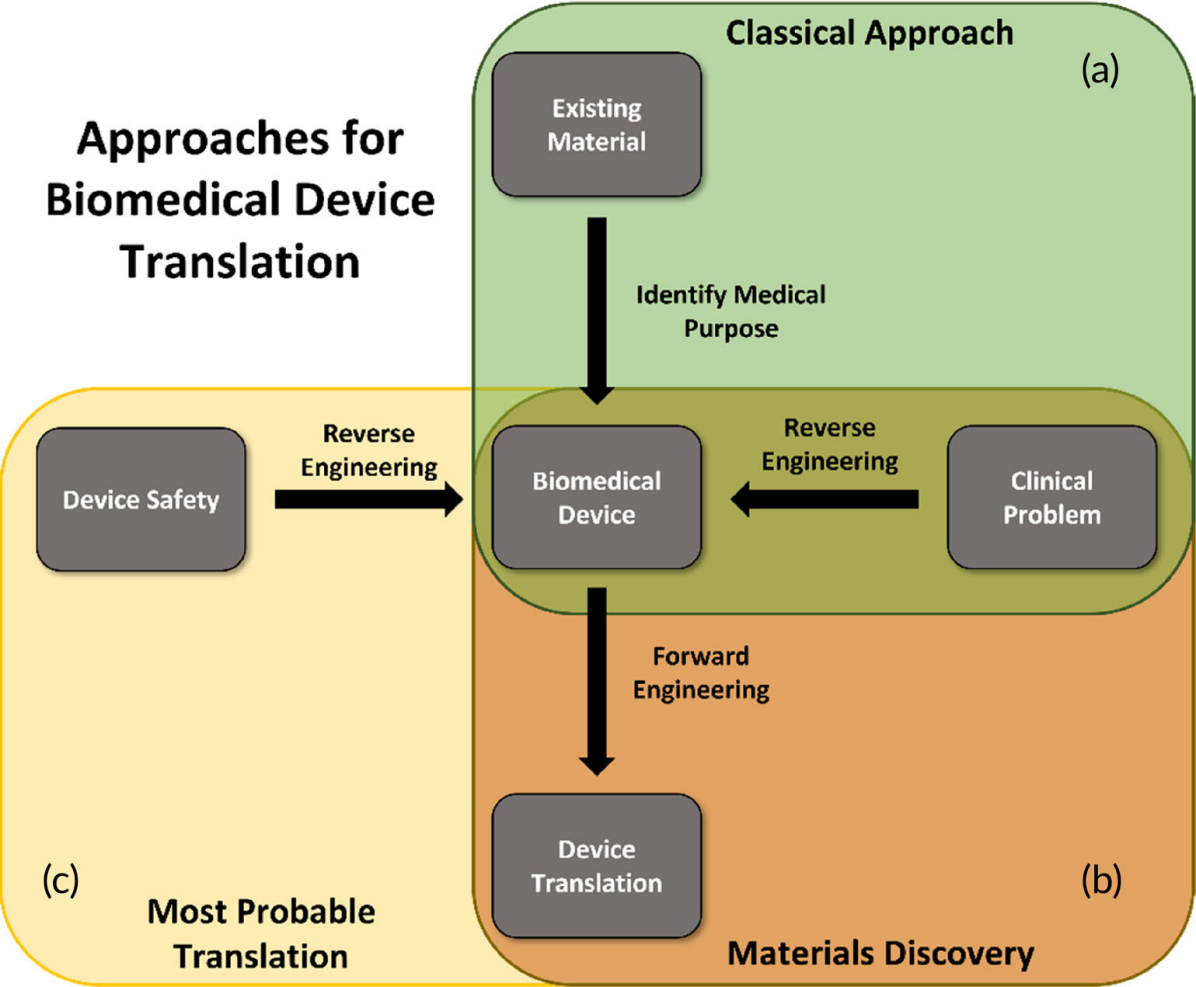
Approaches in biomedical device translation. Design from a clinical problem, using existing materials (a—classical approach—green), design from a clinical problem, using newly discovered materials (b—materials discovery—orange) and safe by design, reverse engineering from a clinical problem, and device safety (c—most probable translation—yellow).

In the 20th century, the use of materials in clinical research was extended to synthetic polymers. As opposed to natural polymers, synthetic polymers often lack properties to promote the differentiation of cells and often require chemical modifications to enhance cellular attachment.^2^ However, synthetic polymers do exhibit physicochemical and mechanical properties resembling those found in biological tissues. Next to this, they are easily fabricated with the possibility to tailor their structure and geometry and allow for controlling their mechanical characteristics while biocompatibility is retained.^24^, ^25^ When used in biomedical applications, biodegradable synthetic polymers are often designed to control, for instance, degradation time, porosity, and mechanical properties. They are cheap since they can be produced in large quantities, and they possess a long shelf‐life.^26^, ^27^ Still, in what may be called the classical mode of biomaterial design, the design process starts with material synthesis, followed by material characterization, and ends with finding the right clinical application for the biomaterial (Figure 2a).^28^ Historically, synthetic materials used in biomedical research were not intended to be specifically used in the human body. Polymers were first synthesized in the lab and have been employed as aircraft windows like poly(methyl methacrylate) PMMA,^29^ whose earliest use as a medical product was noted in 1938 for studying cranial defects in monkeys. Since then, PMMA has been widely used as a material for bone defect repairs.^30^ Polylactic acid (PLA) was initially utilized for rigid packaging containers or bottles before being recognized with excellent bio‐resorption potential and mechanical strength that is in favor of inducing healing processes.^31^, ^32^ In 2010, the PLA‐based stent ABSORB received the CE‐Mark and became the world's first drug‐eluting bioresorbable stent.^33^ Expandable polytetrafluoroethylene (ePTFE), discovered in 1969, has become a widely known anti‐adhesive layer utilized as a coating for frying pans or rain jackets prior to being used in clinics. Despite the very hydrophobic nature of the material, the surface modification of ePTFE by altering the surface chemistry, wettability, or roughness increases the material's biocompatibility and has enabled ePTFE to become an attractive material in biomedical applications, such as stent grafts.^34^

### 2.2 Current approaches in biomaterial design

The design of materials in the biomedical field has started to deviate from using the materials of convenience in clinical research to designing the material for a specific application. Additionally, the lack of autonomous regeneration in humans and the desire to regenerate diseased human tissue gave rise to the new field of tissue engineering and regenerative medicine in the early 1990s, which put an even bigger emphasis on supporting material‐tissue interactions.^35^ One of the leading proponents in the field, Robert Langer, proposed the modern requirements for the scaffolds that were intended to enter the clinics. The new scaffold material should be (1) developed from the biocompatible material that resembles the extracellular matrix and should be easily adapting the desired shape; (2) tested with cells of interest; (3) adapted to the 3D microenvironment observed in the human body; (4) validated by in vitro and in vivo assays, using quantitative molecular and histological assays.^35^

#### 2.2.1 In vitro biomaterial design

Following the classical approach, tissue‐biomaterial interfacial interactions are often underestimated or even neglected, leading to adverse effects such as implant loosening and bad integration.^36^ Also, immune‐related complications, such as inflammation and foreign body response, frequently arise, though still investigated in a trial‐and‐error approach.^37^ A new approach, however, aims at reverse engineering from the clinical problem (Figure 2b), taking a closer look into what the solution and material should be composed of in terms of physicochemical properties to gain optimal host tissue‐biomaterial interaction and implant functioning. Thorough investigation and reverse engineering of currently marketed biomaterial‐based devices, as well as rejected and withdrawn products, inspires the design and synthesis of new types of biomaterials with potentially better clinical success. Some of the pioneers in the field started developing in vitro high throughput screening (HTS) platforms of high complexity but with a low effort of use. These platforms include the screening of multiple complex combinations of material properties to a specific biological response, which in a quick and cost‐effective manner, allows researchers to identify optimal biomaterial design characteristics with minimum effort, all aimed at new biomaterials discovery.^37^, ^38^, ^39^, ^40^ Polymer arrays,^41^, ^42^, ^43^ topographical libraries,^44^, ^45^, ^46^ and multiple physicochemical parameter combinations^47^, ^48^, ^49^, ^50^ are some of the technologies developed in this field of “materiobiology.”^51^ Application of these HTS platforms shifts the biomaterial and medical implant design thinking process from finding a *known* material to tackle the clinical problem to actually discovering *new* materials, reverse‐engineered from the clinical problem, and host tissue‐biomaterial interactions (Figure 2b). This approach may lead to a head start in medical device development early in the process and will ensure better clinical success in the end stages, as compared to more classical routes.^37^

#### 2.2.2 In silico screening and development of biomaterials

Computer‐based design and testing of biomaterials is another new strategy focused mainly on saving efforts for the R&D pipeline process by predicting biomaterial characteristics and interactions.^52^, ^53^ These systematic approaches asses materials using their physical properties to generate a “Digital Twin” and help researchers in the decision‐making process by narrowing the targeted possibilities for the R&D of medical products.^53^ In silico testing is fast and cost‐efficient but requires very complex computational predictive systems and large databases to train them.^54^

The current improvements in deep learning, neuronal networks, and AI have greatly impacted the development of new in silico models. This has enhanced the testing quality for medical product development (like 4D dynamic systems and in silico clinical trials).^55^ Additionally, in silico testing strategies have become crucial for applying safe and/or quality‐by‐design principles.^53^

Due to the vital role of in silico methods in biomaterial development, they have been included as part of the standard requirements of the regulatory agencies in the approval process of medical products.^56^ However, these trends impose an additional endeavor for the regulatory agencies due to the requirement of adequate validation of the in silico models.

The most important in silico methods for biomaterial screening and discovery are, among others, (1) materiomics, (2) automated topography analysis, and (3) computer material–cell interaction modeling.^57^, ^58^ The scientific community is making substantial efforts to make in silico strategies widely available, allowing all scientists to join efforts to improve and validate methods, which will shape the development and evaluation of the future generation of biomaterials.^53^, ^59^, ^60^, ^61^, ^62^

### 2.3 Clinical translation of biomaterials

In parallel to the shift in device design from using existing materials to discovering new materials specifically for medical purposes, the consideration of the biological performance at the early stages of the biomaterial‐based device development, together with the emphasis on the safety aspects of the biomaterial, should drive the overall design requirements for its clinical success (Figure 2c). Therefore, we suggest that a successful clinical translation can only be achieved by close monitoring of the market situation, a good understanding of the market demand, and recognition of new technologies that guide biomaterials discovery through safe‐by‐design principles.

Although an accelerated development of tissue‐engineered constructs is observed, the translation of scaffold materials to the clinic has been extremely limited.^63^, ^64^ This can be attributed to more strict regulatory approvals that are required to bring the more complex biomaterials to clinical practice. Logically, a better comprehension of material–host tissue interactions leads to increased complexity in material design with more specified and controlled biomaterial–host interactions as a main goal. Although tailoring the biomaterial–host interaction is highly important, it does inhibit the transfer and applicability towards the clinic. One of the other reasons for limited device approval is that often, more complex engineered materials are considered as new materials, necessitating the approval process from the start, which is costly and time‐consuming. Additionally, the more sophisticated design of the materials gives rise to concerns in terms of fabrication costs and the number of quality‐control steps.^65^ These developments are among the reasons why the translation of biomaterials and medical devices into the clinic is stagnating. Thus, the current technological advancements in the biomaterials field are, for a part, held back by lagging regulations and show a need to push those forward to speed up clinical translation and prevent further stagnation. Efforts to increase translation to the clinic should include using powerful prediction tools to find an optimum biomaterial design that would support the desired biological outcome.

The paradigm shift observed in the medical device design process leads to devices of increased complexity and more stringent risk assessments. Before marketing a newly developed biomaterial‐based device, a manufacturer should understand and recognize the current nomenclature and risk classification.

## 3 BIOMATERIALS AND MEDICAL DEVICES—NOMENCLATURE AND EU CLASSIFICATION

An important step in the marketing and commercialization of biomaterial‐based devices in Europe is a strict safety and quality assessment known as the CE marking process. CE is a conformity mark that ensures that the safety and legal provisions are fulfilled by the product.^66^ The granting of the CE mark is dependent on the characteristics of the medical device, its intended use, and risk assessment. Without a CE mark, the product cannot be sold and put into service on the EU market.

### 3.1 Medical devices classification

Medical devices are defined by European Legislation as an “instrument, apparatus, appliance, software, implant, reagent, material or other article intended by the manufacturer to be used, alone or in combination, for human beings” and which exhibits one or more specific medical purposes, such as diagnosis, monitoring, prediction, treatment of a disease, among others (listed in detail in Article 2—Definitions.^67^) The definition does specify that medical devices cannot fulfill their medical purpose through the “pharmacological, immunological, or metabolic” paths, as they can only assist in such functions. However, if they do more than assist, such devices would fall under the classification and investigation of the European Medicines Agency (EMA).

As of May 26, 2021, the 1998 Directive 98/79/EC of the European Parliament and the Council on in vitro diagnostic medical devices (IVDD),^68^ the 1993 Council Directive 93/42/EEC on medical devices (MDD),^69^ and the first 1990 Council Directive 90/385/EEC on active implantable medical devices (AIMDD)^70^ were updated with the current device‐related regulations to Regulation (EU) 2017/745 on medical devices (MDR)^67^ and Regulation (EU) 2017/746 on in vitro diagnostic medical devices (IVDR, in force since May 26, 2022).^71^ The MDR recognizes medical device nomenclature and defines medical devices as:

- ‘non‐invasive’ devices,
- ‘active devices’ utilizing the source of energy for their use; software is also included in this category,
- ‘implantable devices’ that intend to enhance the function of certain body parts or to replace damaged tissue,
- ‘invasive devices’ the function of which lies inside the human body.

The claims for the performance of the medical device as intended by its manufacturer imply the medical device classification and further assessment steps. Additionally, there are classification rules (stated in Annex VIII^67^) that should be considered when assessing a biomaterial‐based medical device, that is:

1. What is the intended time of usage? Less than 60 min or more than 30 days? The length of use divides medical devices into transient, short‐term, and long‐term use.
2. Is the device an invasive or an active device? If invasive, how does it enter the human body? The different penetration routes imply specific classifications of a device.

A crucial step in biomaterial‐based product development is the risk assessment. By considering all possible risks and dangers that the device can cause, the safety of patients and the environment can be easily recognized. The standard requirements for the risk evaluation are gathered under international standards ISO 14971 “Application of risk management to medical devices.” Alongside this, the European Medical Device Regulation (MDR) require a Risk Management Plan as an integrated part of the Clinical Investigation Plan. The risk classification of biomaterial‐based products is based on the perceived risks that the medical device might potentially have on the human body, such as the area of contact and the implantation site of the medical device. Additionally, the classification determines the responsibilities of the manufacturers and Notified Bodies (NBs) in a legal framework. NBs are independent public or private organizations authorized by the National Competent Authority and the European Commission to evaluate and certify MDs and IVDs before being implemented.

In the MDR, the risk class of a medical device defines the certification process and post‐market activities. Article 52 in Chapter V of the MDR outlines the conformity assessment procedures for various classes of medical devices, whereas Article 54 specifies conditions under which clinical evaluation and investigation are required. The CE certification process becomes increasingly stringent for higher risk classes, requiring more comprehensive documentation and thorough review by an NB. The post‐market activities are detailed in Chapter VII of the MDR. The articles outline the responsibilities of manufacturers concerning post‐market surveillance (PMS) systems for class I medical devices (Article 85) and periodic safety update reports (PSURs) for medical devices class IIa, IIb, and III.

**FIGURE 3:**
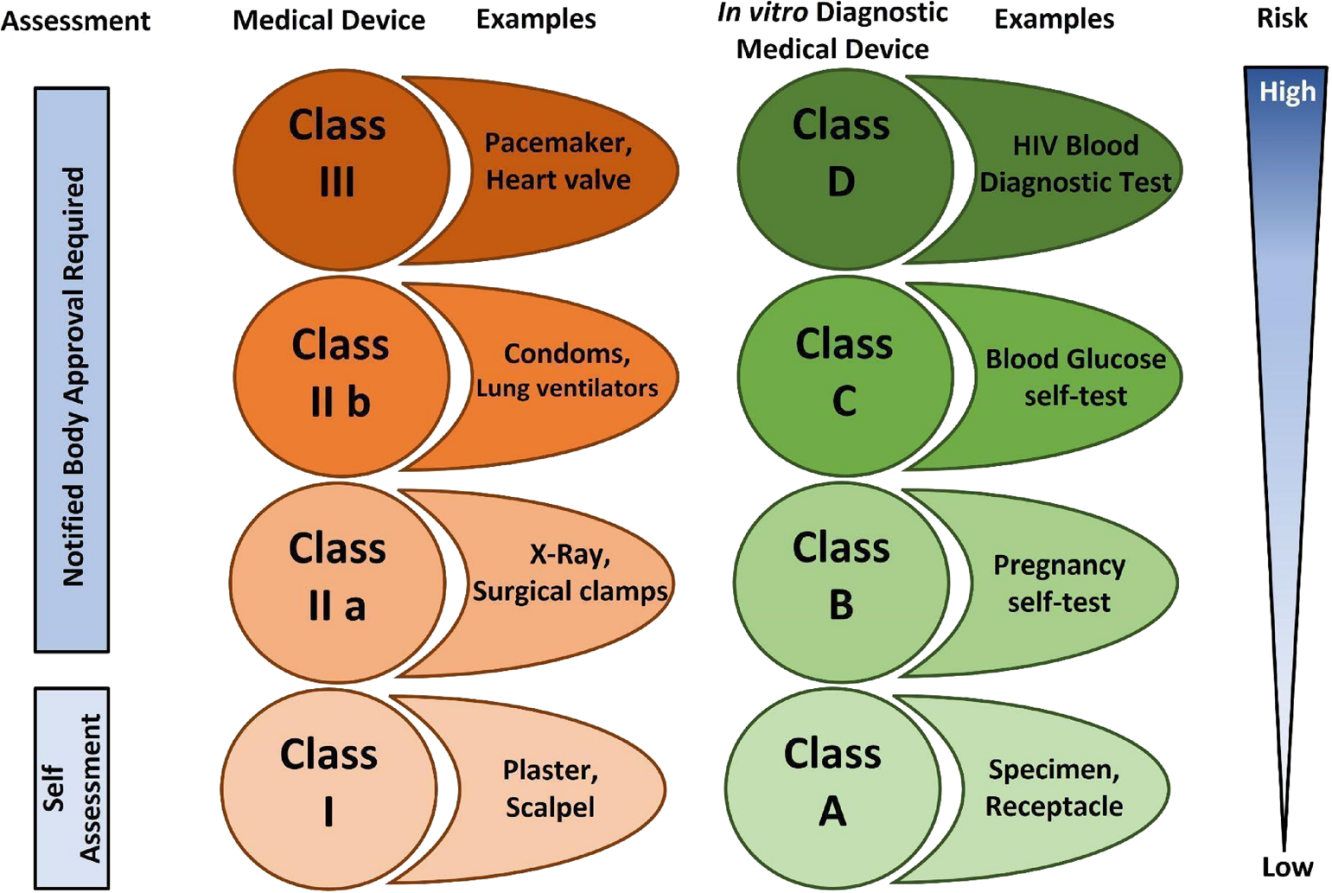
Classification of medical devices and in vitro diagnostic medical devices based on their potential risk to patients.

Based on the risk assessment of a device, the MDR distinguishes four classes of devices: I, IIa, IIb, and III (Figure 3).

- Class I: has the lowest perceived risk, is not life‐supporting, and does not require a NB in the approval process. In this category fall all non‐invasive devices, unless stated otherwise in Annex VIII—Chapter III. Among others, products such as bandages and scalpels are included. (Furthermore, the MDR recognizes four sub‐groups of Class I medical devices: Class I‐non‐sterile; Class Is—sterile; Class Im‐with measuring function; Class Ir‐reusable).^72^
- Class IIa: posing a moderate risk and is mostly used to diagnose or monitor the health conditions of patients. This includes, for instance, thermometers and ultrasonic equipment. All non‐invasive devices that serve as a storage for blood or other body liquids, non‐invasive devices that are in direct contact with injured skin or mucous membranes, surgically invasive devices designated for transient and short‐term use, active therapeutics, software dedicated to monitoring physiological changes in patients and imaging devices using X‐ray radiation fall in this category, unless they have other specifications listed in Annex VIII.^67^
- Class IIb: medium‐risk invasive/active devices, that is, all implantable devices, surgically invasive devices for long‐term use, and devices composed of substance(s) that is(are) dedicated to act locally (skin‐contact) or to be spread throughout the body (inserted into the body via a body orifice—exceptions listed in Annex VIII,^67^ such as lung ventilators).
- Class III: features devices of the highest possible risk, which require permanent monitoring, such as vascular implants or heart‐valve replacements.^67^ All active therapeutic devices that have a built‐in monitoring system providing instant information on patient health conditions, as well as devices comprised of human and/or animal tissue/ cell origin, are assigned to risk class III medical devices.

Additionally, Annex XVI in the MDR categorizes products without a medical purpose. This includes contact lenses, products partially intended for surgical introduction into the human body, substances, or combinations for facial or dermal filling via injection, equipment for reducing or removing adipose tissue, high‐intensity electromagnetic radiation devices (like lasers) for use on the human body, and brain stimulation equipment that uses electrical currents or magnetic fields to modify neuronal activity.^67^

The accurate classification is the manufacturer's responsibility besides following the General Safety and Performance Requirements (GSPRs).^73^, ^74^ The GSPRs, detailed in Annex I of the MDR, set the essential criteria that medical devices must meet to ensure safety and performance before market entry. The GSPRs encompass general safety principles, specific design and manufacturing standards, performance criteria, information requirements, and conditions for certain device types. Furthermore, the manufacturer of the device is subjected to closely monitor the device's performance and present it to the notified bodies (NBs) during device assessment, if needed. If device certification requires it, NBs are there to check and confirm the manufacturer's proposed classification (further specified in Chapter V Article 53), advise the certification route, and estimate the associated costs for the device's CE‐marking.^75^ If the medical device is used in combination with medicines, with ancillary medicinal substances, or with substances that are systemically absorbed, or the device is of high risk, the device will be classified as class IIa, IIb, or III, based on point 5.2 of Annex IX (Rule 14). If this is the case, EMA might participate in the regulatory process (or a competent authority designated by the Member States) and could be consulted by notified bodies (NBs) during the assessment procedures.^76^

Combination products are considered as notoriously difficult to bring to the market. Depending on what component is the most important, either the device or the medicinal product, the MDR or the medicinal regulation like the CTR (Clinical Trials Regulation) is the leading regulation, respectively. For instance, in case the device is primarily designed to provide a mechanical action to open a blood vessel (e.g., a stent) but also to release a drug to prevent blood clotting, the MDR prevails. The drug, in that case, should be an authorized drug. Otherwise, the combination device should also, on top of the MDR, be investigated following the CTR. The route along both the MDR and the CTR is a route that not many manufacturers will take. Advanced therapy medicinal products (ATMPs), which were established due to recent scientific progress in molecular biotechnology, are characterized as either consisting of/or containing recombinant nucleic acids or engineered cells and/or tissue. ATMPs can be combined with a medical device and, as such, serve as an integrated product. The process of clinical transition of ATMPs requires extended preclinical testing and clinical investigations since ATMPs are often associated with the advancement and/or interference with genetic material. In this case, the Committee of Advanced Therapies (CAT) holds the responsibility to prepare a draft opinion on the marketing authorization.

### 3.2 In vitro diagnostic devices classification

Although the definition of biomaterials refers to use inside the body, the field also characterizes a biomaterial as “intended to interact with biological systems.”^4^ As such, another category of biomaterial‐based products is found in “in vitro diagnostic devices” (IVDs).^71^ These devices measure or test human biological samples, for example, blood or urine, to detect or diagnose an individual's medical condition. There is a great variety of IVDs on the market, ranging from blood glucose measuring devices and pregnancy self‐tests to tests determining blood types, HIV tests, and COVID‐19 tests. Similarly, to medical devices, IVDs require a CE mark. However, they do follow a different regulatory framework, which is presented in the document (EU) 2017/746 of the European Parliament.^71^ One of the main reasons behind this differentiation is that, unlike medical devices or pharmaceuticals, the IVD testing devices themselves do not come in contact with the individual taking the test (a swab or sample is taken before diagnosis takes place). Next to this, IVDs only deliver information about the condition and functioning of the body and do not provide any treatment. Although the devices are classified as “not causing direct harm”, incorrect use itself and potentially faulty diagnoses could pose risks to their users.

The IVD industry employs around 75,000 people in Europe and generates an estimated €11 billion in revenue annually. There are approximately 40,000 IVDs in the European market, contributing to reduced total annual healthcare costs by enabling more precise and efficient medical treatments after diagnosis.^71^ Under the old IVD Directive, many IVDs fell into the self‐certifiable group (80% of all IVDs), which did not require NB involvement.^77^, ^78^ The other 20% did require approval by a NB. Under the new regulations, this division is shifted to the other side; only 20% falls into the self‐certifiable group, and the other 80% now do need NB approval in order to stay on the European market.^78^, ^79^ The reason is that IVDs used to be classified based on the type of device. With the new IVDR, similarly to medical devices, IVDs are subdivided into several risk classes (Figure 3), and many of them will fall into the classes requiring NB approval.

Under the IVDR, the IVDs are categorized into four risk classes (A–D). Some examples of devices throughout the different classes^80^ are:

- Products for general laboratory use, buffer solutions, general culture media, histological strains, instruments for IVD procedures, and specimen receptacles.
- Self‐testing devices for detecting pregnancy, fertility, cholesterol levels, glucose, erythrocytes, leukocytes, and bacteria in urine. In this category fall all other IVDs not otherwise classified and devices that serve as controls without a quantitative or qualitative assigned value.
- Devices intended to test for blood grouping or tissue typing for other markers, devices intended to detect the presence of some infectious agents, companion diagnostics, infective disease status, disease staging, genetic testing, congenital disorder screening, and others; all other self‐testing devices not listed fall in category B.
- IVDs are used to detect the presence of transmissible agents in blood and organs to assess their suitability for transfusion, blood grouping, or tissue typing tests used to determine markers for the ABO system, Rhesus, Kell, Kidd, and Duffy systems.

Determining which device falls into which risk class is based on a set of seven rules in Annex VIII of the IVDR.^71^ In contrast to the Directive, where IVDs were classified in lists according to their type, the Regulation categorizes IVDs into four risk classes based on perceived risk for the end‐user and the broader community. In the new scheme of biomaterial‐based device conformity assessment, focus is put on the safety of the device to users, and depending on the risk of both medical devices and in vitro diagnostic devices, the relevant clinical assessment must be conducted and evaluated before placing the device on the European market.

### 3.3 In‐house manufactured devices

In‐house (IH) manufactured devices fall under Article 5.5 of both the IVDR and MDR (besides being mentioned in Recital 30 of the MDR, and 29 of the IVDR).^81^ This means that devices manufactured and used within health institutions do not need to undergo the CE‐marking process. Previous regulations (MDD and IVDD) lacked proper guidelines for in vitro tests and the production of MDs and IVDDs in a non‐commercial context. In contrast, the new regulations (MDR and IVDR) limit the use and application of IH devices. Developing an IH device can be a strategy to not have to go through the lengthy and costly process of CE‐marking, though clinical impact with the device will be limited, as it can only be used in the legal entity in which it is developed.

## 4 EUROPEAN DEVICE REGULATIONS—PROCEDURE FROM BENCH TO BEDSIDE

In the first quarter of 2023, the European market evidenced an approximate 23.1% increase in medical device recall compared the average of all quarters of the last 3 years.^82^ This trend poses a significant endeavor for the new MD—IVD regulations and European market stakeholders. Hence, it shows the importance of post market studies, and that the clinical success of medical devices is, by no means, guaranteed by the MD approval process alone. This entailed an update of clinical evaluations and regulations to align with the requirements of the evolving medical device market. The EU reflected upon the situation over the last 20 years, observing the regulation blind spots and declining trend of device approval, and has revised the legal frameworks. Also, by changing from directives to regulations, both MDR and IVDR became direct legislation in the member states (MS) within the EU.

### 4.1 Low biomaterials‐based devices approval rate in Europe (MDR)

Historically the preference for introducing new medical technologies leaned towards the CE mark route due to its cost‐effectiveness and faster process. However, an analysis conducted by UCLA Biodesign on market approvals of innovations between 2010 and 2022 revealed a shift in favor of the US market. With the introduction of the MDR, the US has now surpassed the EU market in terms of successful approval rates.^83^ The American Food and Drug Administration (FDA) holds a reputation for keeping up with the pace of advancing technology and manages more efficiently the implementation process into the market.

Numerous regulatory scandals surrounding breast implants,^84^ as well as cases involving hip‐on‐metal implants,^85^ and birth controls,^86^ have caused irreversible consequences for patient's health and quality of life, leading to the reappraisal of the medical device risk classifications. These regulatory failures and their associated negative outcomes emphasized the need to prioritize human safety and triggered a profound change in the EU medical devices field, resulting in the new MDR.^67^

By introducing the MDR, the EU intended to provide a new level of safety and quality in the medical device field. Quite similar to the previous directives, the new regulations hold the same basic requirements but are more stringent in some aspects, especially in terms of risk classes. One of the most important additions to the regulations is that, for the first time in the EU, the MDR raises the topic of registries.^86^ Registries should be formulated for certain technologies that would lead to a more homogenous information collection between MSs. It requires both the manufacturers and the NBs to ensure that post‐market surveillance is accessible for each device that has entered the market.^87^ With the new legislation, the EU improves transparency through a European Database on Medical Devices (EUDAMED, mandatory starting not earlier than 2024) and a device traceability system based on unique device identification (UDI), which allows continuous monitoring and verification of the specific commodity on the EU market (IH devices are exempted from UDI requirement).^88^, ^89^ These new tools are especially important for clinicians, patients, and stakeholders to foresee the decision‐making process of implementing a novel treatment.

Another reason for implementing the MDR is that in the past, across the EU, many countries applied their own set of rules for handling the CE marking process.^87^ This mostly referred to a biomaterial‐based device's technical aspects instead of the clinical investigation design and safety testing procedures. Up to 2017, across the EU countries, clear and uniform guidelines on the clinical trials protocol and CE marking of biomaterial‐based devices were lacking. For many years this has provoked companies to operate in locally concentrated areas rather than throughout the EU. This resulted in increased flexibility for individual EU countries to adjust clinical investigation plans (CIPs), which are essential for the successful clinical translation of a medical device. CIPs identify the rationale for the clinical investigations, including risk assessment and the acceptance criteria, and give the fundamentals for the clinical investigation conduct.^90^ Despite a set of guidelines for the clinical investigation of medical devices for human subjects (ISO 14155), the country‐specific regulations and legislations decrease the quality of EU inspections and, thus, medical device approval.^87^, ^91^

**FIGURE 4:**
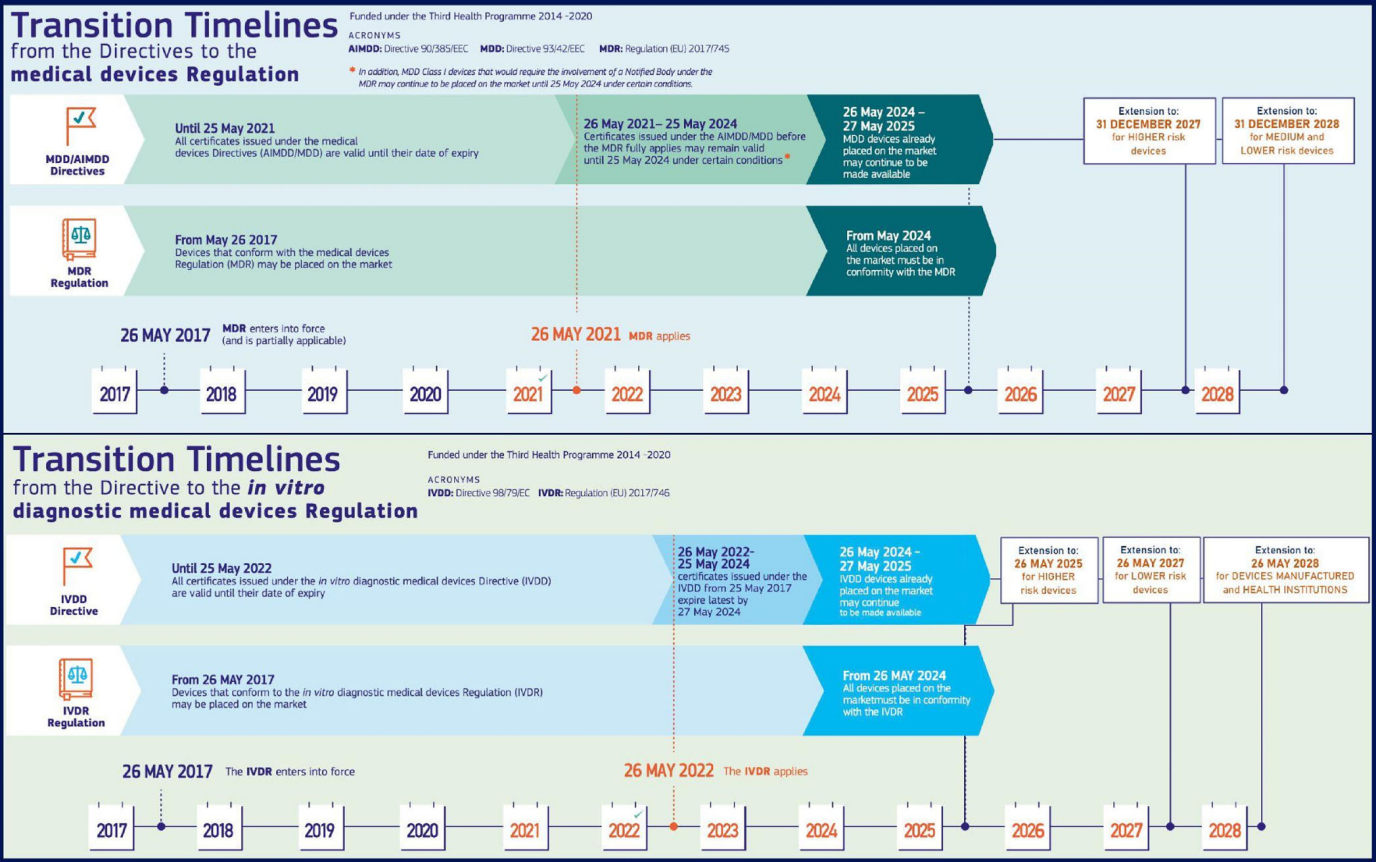
Transition timelines figure based from the Directives (2017) to the Regulations (2025) (*Source*: Figure adapted and modified from European Medicines Agency timelines)^92^ extended with updated deadlines by the Council of the European Union.^93^

Recently, the European Medical Device legislation has been transformed. Manufacturers now need to comply with the new regulations MDR from 2022 onwards. The European Union has provided some useful information on the transition timelines (Figure 4).

While introducing the MDR will be beneficial for safety and EU‐broad transparency, the execution and implementation of the MDR raises several concerns. Even though the European Clinical Trial Tracker was launched in 2018 and has already reported a 25% increase in the number of clinical trial records, it will take a long time for the EUDAMED to be fully functional and up to date within the EU.^94^ Another concern lies in conformity assessment and NBs. While the number of NBs declined in the past, as a result of stringent rules on their performance, their workload had substantially risen.^91^, ^95^ Currently, there are a total of 49 notified bodies throughout the whole European Union (49 under MDR and 12 under IVDR updated July 2024), with approximately 8000 new applications under the MDR and issued 2000 CE certificates.^96^, ^97^ The MDR demands physical inspections, a solid quality management system (QMS), and the performance of laboratory tests on medical devices by NBs during a clinical and ethical product assessments. Furthermore, the NB's competences need to undergo regular verification by an accreditation organization to guarantee ethical standards within the EU and prevent future misconduct.^91^ In addition, the transition period requires medical device companies to change their existing MDD certification to a newer version that is compliant with the new MDR. Their current technical product documentation (TPD) needs to be in compliance with the MDR, and their QMS needs to comply with ISO 13485. If the certification is not renewed in time, the actual CE marks assigned by NBs will no longer allow for the medical device trade within the EU.^98^ Therefore, the important step towards advancing validation methodology may as well pose substantial difficulties for many stakeholders in the med‐tech sector. Additionally, it creates a huge gap in providing CE‐marks until all the changes are fully adapted and distributed among responsible parties.

The new MDR also introduced some products used for esthetic treatments as medical devices, such as injectable substances (cosmetic fillers), which in some cases could also be perceived as having a treatment effect on patients or containing ancillary medical products classified as Class III devices. The MDR classifications could be perceived as a vast improvement in reclassifying some products for esthetic treatments that, in other cases, would not have been highly regulated. However, some of the devices without medical purpose could lead to serious safety concerns, especially towards new evidence of immunological side‐effects (e.g., autoimmune/inflammatory syndrome induced by adjuvants [ASIA] and large cell lymphoma) that still need a close investigation and could need a more robust pharmaceutical approval process.^99^ This modification in the MDR addresses a big gap in the regulation of esthetic treatments that have great significance in the market nowadays.

**FIGURE 5:**
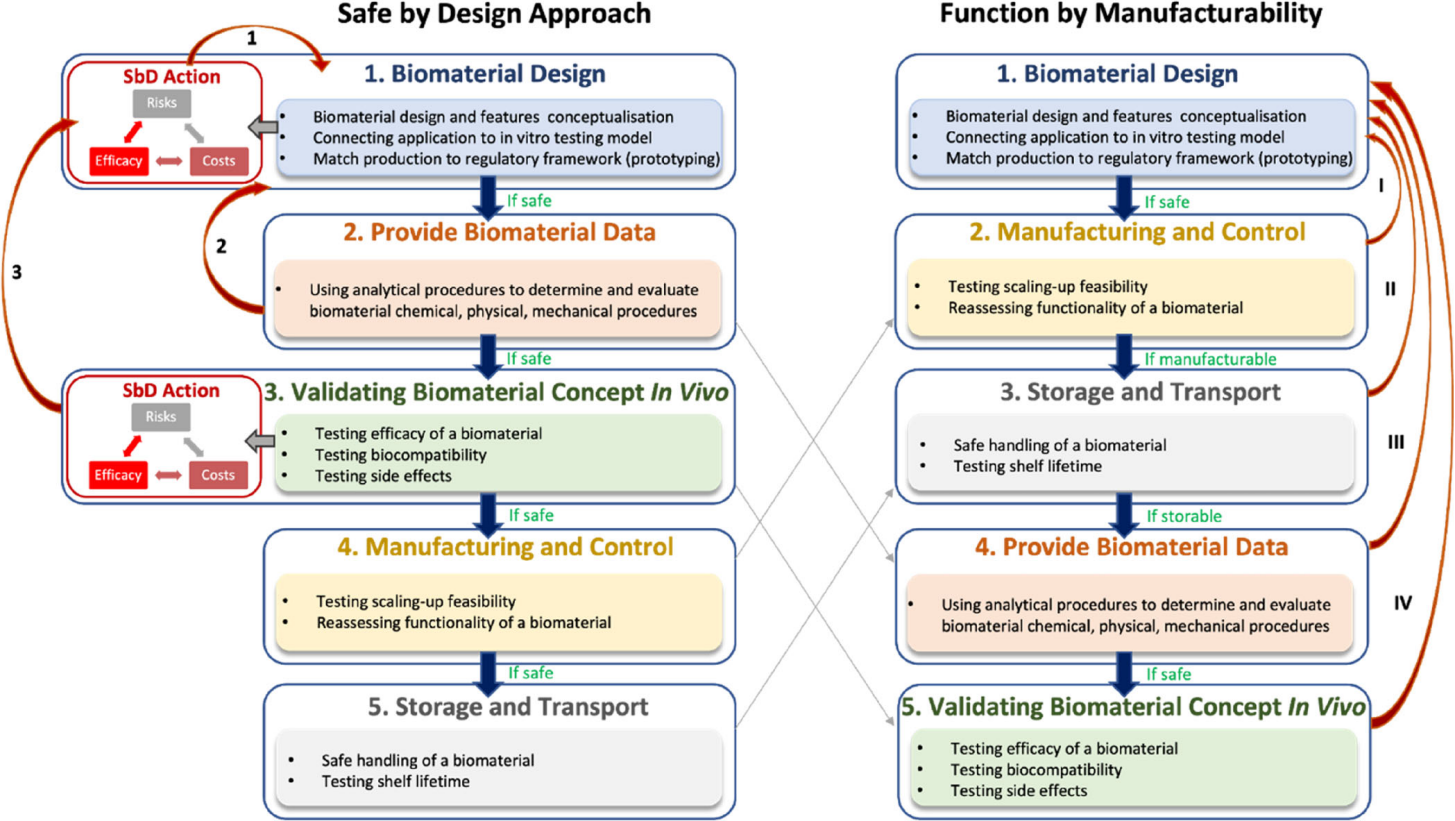
The safe by design approach working scheme, together with the proposed function by manufacturability strategy for biomaterial‐based medical device design. The red arrows in the SbD approach (1, 2, and 3) indicate the feedback loops that should be considered when the biomaterial‐based medical device is unsafe. The red arrows in the FbM approach indicate the feedback loops that should be applied when the biomaterial‐based device is unsafe (I), non‐manufacturable (II), non‐storable (III), or unsafe to be validated in vivo (IV). The gray arrows show the suggested shift in the workflow of function by manufacturability strategy. Inspired by Som et al.^133^

Furthermore, claiming equivalence in a medical device approval process was a big part of the MD approval rate and market introduction arguments. The MDR introduced stricter criteria for applying clinical, technical, and biological equivalence of medical devices (especially in class III devices).^100^ Additionally, a scrutiny process has been included in the MDR for medical devices class III and IIb to optimize the process done by NB to high‐risk and innovative medical devices by performing a highly detailed examination to ensure that all regulatory requirements are met. These measurements are expected to improve the safety and efficacy of newly approved medical devices because a more significant portion of them should go through the pre and post market clinical investigations. In the MDR, the medical device classification and the risk level (also described by ISO 14971) are redefined and will help foresee the Quality Assurance and Regulatory Affairs (QA/RA) pathway, allowing the development of an appropriate investigation plan.^90^ Thus, essential approaches such as “safe by design” (SbD) at the beginning of the medical device design,^101^, ^102^ combining R&D with safety, quality, and efficacy assessment throughout the MD development, will lead to potentially reducing the growing medical device recall rate (Figure 5).

We are introducing another perspective that emphasizes translational research and ethical consideration, which is the “function by manufacturability” (FbM) approach.^101^, ^102^, ^103^, ^104^, ^105^ This approach aims to first test the manufacturing feasibility and scalability of biomaterial‐based medical devices before conducting in vivo evaluations. In this way, the bench‐to‐bedside pathway minimizes reliance on animal testing and allows researchers to determine first whether the biomaterial design can be safely produced. Overall, the advancements are intended to improve the safety of users and to validate biomaterials‐based products that play a crucial role in numerous therapies and healing processes. Both approaches, SbD and FbM, might be implemented at the start of the biomaterial development for medical device purposes to ensure smooth clinical translation. As mentioned, the MDR aims to create a uniform legislation framework across the EU. Nevertheless, the MDR may deprioritize the EU market due to its stricter expectations and requirements for a medical device's approval. Since the CE Mark process becomes more complex, stakeholders are driven to favor different markets, such as the US, due to its clarity in regulatory pathways and smooth adaptation to new technologies.^83^ It is being speculated that the same scenario of tightening medical device regulatory approval may happen to other metrics soon.

### 4.2 In‐vitro diagnostics regulations

Similar to the case of the MDR implementation, the In Vitro Diagnostic Directive (IVDD) has changed to the IVD regulations (IVDR) and must follow the same timetable (Figure 4). The EU emphasizes the importance of an early start of adaptation to the new regulations. The pressure put on NBs during the transition period is tremendous, especially concerning the increased workload and assuring the quality of their work on device inspections.^95^ As an example, a huge concern about envisioned bottlenecks was already put forward by the Dutch National Institute for Public Health and the Environment (RIVM) in 2018.^79^ They assessed the distribution of IVDs registered in the Netherlands over the different risk classes of the new IVDR and the shift from the categories of the IVDD to the IVDR classes A–D (Figure 3). Their main conclusions encompass that under the IVDR, more IVDs require a notified body approval in order to obtain market access. The assessment and classification of a random sample of all IVDs registered in the Netherlands showed that under the IVDD, 7% of the IVDs require assessment by an NB. However, under the new IVDR, up to 84% of the IVDs that are currently registered in the Netherlands will require an NB assessment for market access.^79^ Lubbers et al. add that even though the old and new legislations share the concept of IVD certification and CE marking, more (clinical) performance data and documentation will be required to legitimately commercialize IVDs under the new IVDR.^106^ The new situation does not only affect manufacturers all over the EU, but also all diagnostic laboratories, be it in‐house or external.^107^

### 4.3 ISO standards in Medical Device Assessment

One of the essential parts of any R&D process should be identifying the regulatory procedures necessary for the device approval and compliance with all the regulatory requests of the specific medical device classification. This evaluation should be done repeatedly throughout the medical device development process (classification and risk assessment loop) in order to avoid hurdles in taking the device to the market.^90^

Thus, in the R&D process, the relationships with fundamental stakeholders (patients, care providers, physicians, policymakers, regulatory agencies, producers, among others) are identified, conceptualized, and planned. Ergo, R&D puts into perspective the decision on the future investment (with the risks) of developing and bringing a medical product onto the market.^108^ For this reason, creating a solid preclinical and clinical investigation plan (CIP) is valuable and fundamental, which can be guided by the ISO standards 10933 and 14155.

ISO 10993 standards were issued as a framework for biological response and biocompatibility of biomaterial‐based medical devices.^109^ Likewise, safety and efficacy assessments are necessary procedures for implementing a medical product into the clinic, which is highly related to the market approval regulations. These steps are mostly known because they include the clinical investigation which are guided by ISO 14155.^110^ These standards are widely accepted and suggested as guidelines by authorities such as EMA and FDA. Nevertheless, biocompatibility lacks a comprehensive review of the ISO framework. For example, the ISO 10993 framework for cytotoxicity assays, which aims to assess the cell reaction to a biomaterial and its leachables, has been reported as failing to successfully evaluate the response of implantable biomaterials due to insufficient testing.^111^, ^112^, ^113^, ^114^, ^115^ Moreover, the ISO standards often do not test specific properties that make a device design innovative (e.g., in antimicrobial surface designs many ISO standards do not make a clear distinction between releasing and non‐not releasing systems). To verify the unique functional advantages of a design, some dedicated tests are needed, on top of the ISO standards.^111^ Furthermore, infections, foreign body response, and fibrotic encapsulation are often neglected in ISO's recommended biocompatibility investigations, which are the leading causes of implant rejection, as revealed by in vivo studies, and are a fundamental source for medical device recall.^116^ Thus, biomaterial‐based medical devices require more specific analyses to demonstrate safety by design so they can (smoothly and successfully) enter the market with a higher success rate.^117^

Hence, it has been suggested that ISO standards are no longer up‐to‐date and do not always relate to safe and successful medical product development.^118^, ^119^, ^120^ Still, finding and applying appropriate, applicable and available ISO standards is often recommended, apart from defining and applying own, better applicable, but still to be validated test methods. Nonetheless, ISO standards remain the leading guidelines for verifying the safety of a medical device before accessing the market to date. Therefore, efforts are being made to improve quality standards that can have a positive impact on reducing medical device recall and withdrawal, besides increasing the number of approved products, such as the revised 10993‐17 updated on 2023 for the “Biological Evaluations of Medical Devices” which includes the toxicological risk assessment of medical devices and its leachables and the recommendation for using several testing methods for evaluation of medical device interactions.^121^, ^122^ However, the impact of the updated 10993‐17 guideline has yet to become clear. Additionally, several standards have been developed in response to the ISO framework limitations, such as by the United States Pharmacopeia (USP) and the American Society for Testing and Materials (ASTM), presenting specific protocols for conducting various biological tests.^123^ Subsequently, in the EU, several in vitro and in vivo tests have been developed to meet the MDR safety and performance requirements.^124^

A revised ISO 10993 framework was released in 2021, and it had high expectations from the field to address the vital standardization issues of previous versions. The 2021 ISO guideline proposes new specific methods to evaluate medical devices, such as skin irritation, and stresses the shift from in vivo to in vitro tests as the preferred models. However, the poor correlation between in vitro tests and in vivo models has been vastly noted by the scientific community.^125^, ^126^ Additionally, the standard still does not cover an exhaustive list of possible materials used within medical devices and direct test approaches for each situation. Then, the new ISO framework could still be improved in some points, enhancing the pertinence of the guidelines, and strengthening them to be a more reliable gold standard. Thus, it could positively impact medical device safety and diminish their market recall and withdrawal.^111^, ^123^

### 4.4 Technological transfer and marketing

The successful route from bench to bedside of biomaterials depends, among others, on the selected area for marketing the product. The choice of the market area approval encompasses the total addressable market, market competition, long‐term predictability of costs for the market launch, difficulty of regulatory processes, and possibility of reimbursement.

On the other hand, two important processes for clinical translation are scalability and technology transfer (TT).^127^, ^128^ The upscaling capability of the biomaterial must be evaluated from different perspectives. First, by assessing the market and the production capacity and assuring that the biomaterial preserves its mechanical properties and biological performance. Subsequently, the production upscaling should aim for minimal hazardous waste risks, considering the environmental impact and safety in the early stages of innovation development. An ISO framework has also been established for these procedures (ISO 13485), ensuring quality management to meet the requirements for regulatory purposes.^129^

With well‐established ideas and product development, the technology transfer process is key for medical device translation. This step bridges the research and development phase with the commercialization and marketing stage and should be considered from the start of product development. Technological transfer centers (TTCs) are a newly introduced strategy in EU countries, encouraging researchers to follow the transition of technology from academia to the market. This is done by securing intellectual property rights, funding, and assessing the market potential for the new technology. Moreover, a technology transfer approach may contribute to building networks between researchers, business representatives, governmental organizations, and venture capitalists. TTCs contribute to the spin‐off creation and facilitate the interaction with stakeholders, which is especially important in the academic field, where money is limited and going through MDR relies on external partners and investors as the process is costly and time‐consuming.^130^

Commercial and marketing challenges are also relevant for medical product success, and approaching medical device translation from a business perspective should not be neglected. Yet, safety and efficacy should be paramount in developing medical devices and must be considered a priority, as stressed in this review (Figure 5). After a device enters the market, continuous clinical evaluation is required. It is crucial to follow advancements and failures in the field of interest to deliver the best possible product. Furthermore, the in vivo screening is continued after the biomaterial enters the market (post‐market evaluation) to monitor long‐term biomaterial side effects (addressed in ISO/TR 20416).^131^ Generation of the so‐called post‐marketing clinical follow‐up (PMCF) is now required for any devices entering the market and is essential to detect any undesirable effects quickly while highlighting areas for improvement and preventing market recall causes.^132^

Taking all into consideration, the success of biomaterials transitioning to the clinic is primarily based on a solid R&D phase with an emphasis on medical device safety assessment, evaluating the clinical needs, and communication with stakeholders. Thus, the first steps of biomaterial development are fundamental to unlocking the market potential and should be the focus of the regulatory agencies and legislation, ensuring an effective market transition without limiting innovation. Additionally, ISO standards are helpful guidelines that can take the stakeholders through all the processes, yet they are not bulletproof, and further references should be consulted for all cases. MDCG (Medical Device Coordination Group) guidelines are also critical resources for understanding the regulations and applying step‐by‐step approval requirements for MDs and IVDs, which take the hurdles of understanding law regulations and eases the adhesion to the rules. Lastly, early preparation and continuous assessments are fundamental components for solid investigation and commercialization plans, leading to a smooth and successful clinical transition of biomaterial‐based medical devices into the clinic.

## 5 CONCLUSIONS AND FUTURE OUTLOOKS

The development and approval of biomaterials for medical use can be a slow and costly process, subject to stringent safety regulations. However, MDR and IVDR represent a significant improvement towards patient safety and healthcare, which is a priority for the European medical device field. In this review, we summarized several key contributing factors in order to gain a better understanding of the transition from MDD to MDR and IVDR and how they affect the translation of biomaterials to use in a medical device in the clinic in the EU. Concluding, we can put forward the following statements:

Throughout the course of the last century, there has been a remarkable evolution in the development of biomaterials for clinical applications. The shift in mindset from using materials out of convenience to discovering actual new materials, and reverse engineering from the clinical need and the patients' safety, ensures better performance and safer products. This shift has led to the exploration of more complex biomaterials with specific functions and uses and has facilitated the development of in vitro and in silico high‐throughput screening platforms to identify optimal parameters for new biomaterial designs. This progression in biomaterial research accelerated the design of medical devices but, at the same time, complicated the approval process.

As biomaterials have become more intricate, concerns regarding their safety have also risen. These concerns have prompted the implementation of stricter requirements for the clinical translation of biomaterials, therefore slowing the process of medical device approval. In Europe, the introduction of the MDR was driven by a focus on patient safety. The MDR emphasizes risk assessment and patient safety in the classification of medical devices. The regulations aim to enhance transparency, safety, and performance of medical devices through rigorous requirements, post‐market surveillance, and the utilization of registries. While ISO standards play a crucial role in the medical device assessment process and the guidelines represent the most suitable instrument for assessing medical device safety, there is still room for improvements concerning their comprehensiveness and relevance in ensuring safety and successful medical device development. We suggest an early implementation of a safe‐by‐design approach, as described in Sections 2 and 4.

Efforts are ongoing to improve quality standards in order to address limitations and increase the number of approved products while reducing the rates of medical device recalls. It is primarily done by preparation of a solid R&D plan and QMS, where medical safety is paramount and clinical needs are evaluated. Promoting and implementing research on safety and quality by design strategies should be prioritized. We suggest that the initial stages of biomaterial development, using safe‐by‐design and/or function‐by‐manufacturability approaches (integrated into the ISO standards, MDR, and IVDR, and followed by correct risk assessment) might be crucial in the future for unlocking market potential and facilitating a smooth transition into the European medical device market.

The transition from the MDD to the MDR and IVDR poses challenges, including increased workload for notified bodies and potentially deprioritizing the EU market for manufacturers due to the complex approval process. Safety and efficacy are prioritized in the new MDR and IVDR. SbD and FbM (Figure 5) approaches prioritize the integration of safety considerations into biomaterial‐based medical device design, ensuring that manufacturing processes are optimized for both functionality and regulatory compliance. We argue that new product development might be prioritized on the application of the MDR and IVDR and extend the time and support for the re‐classification of established devices to keep bringing patient care solutions to the European market instead of re‐marketing potentially outdated medical devices. This could prevent established medical devices from being discontinued because of bureaucracy and/or financial reasons, allowing patients to keep receiving imperatively needed treatments and avoiding the saturation of NBs. Moreover, by identifying how both medical device developments and changing European regulations relate to one another in a clinical translational context, we envision to assist researchers and medical device developers (besides other stakeholders) in following a smoother and more successful path to clinical translation within the European market.

## AUTHOR CONTRIBUTIONS

**Klaudia M. Jurczak:** Conceptualization; writing – original draft. **Torben A. B. van der Boon:** Conceptualization; writing – original draft. **Raul Devia‐Rodriguez:** Conceptualization; writing – original draft. **Richte C. L. Schuurmann:** Writing – review and editing; supervision; conceptualization. **Jelmer Sjollema:** Writing – review and editing; conceptualization. **Lidia van Huizen:** Conceptualization; writing – review and editing. **Jean‐Paul P. M. De Vries:** Conceptualization; writing – review and editing; supervision; funding acquisition. **Patrick van Rijn:** Conceptualization; funding acquisition; writing – review and editing; supervision.

## FUNDING INFORMATION

This review was financially supported by Jaap Schouten Foundation, co‐financed by SNN (Samenwerkingsverband Noord‐Nederland, Northern Netherlands Provinces Alliance:OPSN0361) and the Graduate School of Medical Sciences (GSMS) at the University of Groningen.

## CONFLICT OF INTEREST STATEMENT

The authors declare the following conflict of interests: P. van Rijn also is co‐founder, scientific advisor, and shareholder of BiomACS BV, a biomedical‐oriented screening company.

### PEER REVIEW

The peer review history for this article is available at https://www.webofscience.com/api/gateway/wos/peer-review/10.1002/btm2.10721.

## Data Availability

Data sharing is not applicable to this article as no new data were created or analyzed in this study.
